# Computational Insights into Selective Water–Methanol Transport in rGO/PSS Composite Films

**DOI:** 10.3390/molecules31101657

**Published:** 2026-05-14

**Authors:** João Felipe da Silva Almeida, Nathan Rabelo Martins, Daiane Damasceno Borges

**Affiliations:** 1 Instituto de Física, Universidade Federal de Uberlândia, Uberlândia 38408-100, MG, Brazil; j.felipe.novem@gmail.com; 2 Instituto de Ciências Exatas e Tecnológicas, Universidade Federal de Viçosa, Campus Rio Paranaíba, Rio Paranaíba 38810-000, MG, Brazil; nathanrabelo@ufv.br; 3 Department of Physics, Texas State University, San Marcos, TX 78666, USA

**Keywords:** selective diffusion, rGO-based composite, molecular dynamics simulation

## Abstract

Reduced graphene oxide (rGO) wrapped with poly(styrenesulfonate) (PSS) forms a stable hybrid material (rGO/PSS) capable of producing ultrathin films with promising barrier properties for Direct Methanol Fuel Cell (DMFC) applications. These films aim to mitigate methanol crossover, one of the major limitations of DMFC technology. In this work, we investigate the mechanisms underlying the methanol barrier effect of rGO/PSS, while maintaining water permeability. Classical Molecular Dynamics simulations were employed to explore the structural and dynamic properties of rGO/PSS at different polymer ionization fractions in a solvent mixture of water, methanol, and hydronium. The influence of the sulfonation fraction on film self-assembly was analyzed, including its impact on PSS conformation, rGO sheet distribution, and PSS–rGO interactions. Finally, the effect of the rGO/PSS structure on solvent diffusion was investigated, and the mechanisms responsible for the selective transport of methanol were elucidated.

## 1. Introduction

Graphene and graphene-related materials have emerged as key components in the development of eco-friendly energy technologies because of their numerous relevant features largely explored by the scientific community in the past decades. In particular, reduced graphene oxide (rGO) is widely explored as a practical alternative to pristine graphene. Unlike graphene, rGO can be produced at a large scale and lower cost via chemical routes, making it more compatible with industrial membrane fabrication. Unfortunately, the strong π–π stacking and van der Waals interactions between graphene sheets promote restacking and agglomeration, resulting in poor colloidal stability in dispersion and, naturally, prevent an easy and straightforward use of this 2-dimensional (2D) material.

Non-covalent functionalization with polyelectrolyte represents an effective strategy to stabilize graphene-based sheets in aqueous media [1]. Poly(styrenesulfonate) (PSS) was among the first stabilizing agent used during the reduction of graphene oxide [2]. This polyelectrolyte stabilizes rGO through sulfonate-induced electrostatic repulsion and π–π-mediated adsorption of its aromatic backbone onto graphene basal planes, providing combined electrostatic and steric stabilization against restacking. Nevertheless, the effectiveness of PSS as a stabilizer depends on polymer properties (molecular weight and charge density) and on preparation conditions (degree of reduction, sonication, and concentration) [3,4].

The formation of stable aqueous rGO/PSS inks enables their use in layer-by-layer (LbL) self-assembly methods, facilitating the fabrication of composite membranes [5]. In particular, the LbL bilayer composed of the cationic polyelectrolyte poly(allylamine hydrochloride) (PAH) and rGO/PSS exhibit promising methanol barrier properties for Direct Methanol Fuel Cell (DMFC) applications. One of the main challenges to DMFC operation is the phenomenon known as methanol crossover, which is characterized by the undesired diffusion of methanol through the polyelectrolyte membrane, resulting in electrolyte poisoning and a drastic reduction in energy efficiency [6]. One strategy to solve this problem is the deposition of ultrathin layers at the anode–electrolyte interface. This physical barrier must be able to block the methanol passage without blocking the water flow and proton conduction [7]. PEDOT:PSS self-assembled films have been used to reduce methanol crossover [8], however, effective methanol blocking was achieved only when graphene oxide was incorporated into the PSS matrix [9,10].

In this context, the objective of the present work is to investigate the mechanism by which rGO/PSS acts as a methanol barrier without reducing the permeation of water. For that, Molecular Dynamics (MD) simulations were performed to investigate the structural and dynamic properties of rGO/PSS, as this technique enables molecular-level analysis of structural behavior and its time evolution. Several studies have employed MD simulations to investigate polymeric systems, including PSS [11,12,13,14]. Recent studies have inferred the limitation at determining equilibrium properties of polymers in solution by brute force atomistic MD simulation [15]. Such simulations pose significant challenges, often requiring enhanced sampling techniques, simplified scenarios [16] or coarse-graining approaches [17].

To accomplish the aim of this study, several MD simulations were performed to examine the structural and dynamic properties of rGO/PSS at different degrees of polymer ionization in a solvent composed of water, methanol, and hydronium ions. Regarding the structural properties, we discussed how the degree of sulfonation influences the self-organization of the layered film, including its effect on the PSS conformation and the distribution of rGO sheets, as well as describing the nature of the interactions between PSS and rGO. Concerning the dynamic properties, the impact of the rGO/PSS structure on solvent diffusion was analyzed, and the mechanisms leading to selective methanol diffusion were elucidated.

## 2. Results and Discussion

The properties of self-organized rGO/PSS thin films were investigated using MD simulations of a system comprising two rGO sheets and eight PSS chains, as detailed in Section 3. Each rGO sheet has an O/C ratio of 0.07 and contains three hole-type defects. We investigated five PSS sulfonation fractions, defined as the ratio of sulfonated groups to the total number of monomers, denoted as: f1, f075, f05A, f05B and f025. For fraction f05, which has half of the chains functionalized with $SO_{3}^{-}$, two configurations were made, in which, in one of them, the $SO_{3}^{-}$ is arranged on the same side of the polymer (f05A), more hydrophobic, and in the other is arranged alternately (f05B), more hydrophilic. The film was equilibrated in a 90:10 water–methanol solution (~18 wt% solvent), with hydronium counterions added to maintain charge neutrality. In result, the simulation boxes have a total number of atoms ranging from 6480 to 7152. More detailed information about the simulations is found in Section 3.

For each composition studied, we have performed 10 different MD simulations starting from a randomly generated initial configuration. During the equilibration, the simulation box was progressively compressed along the z-direction to obtain a bulk-like thin film geometry, while allowing the system to relax toward thermodynamic equilibrium. In result, the simulation box sizes vary around approximately 63 × 61 × 18.5–20 Å. At this stage, different initial configurations evolved into distinct relaxation structures, indicating a strong dependence of the final state on the initial configuration. Although the total energy reached a common value (Figure S1), one would expect that all configurations with the same composition converge to the same final state of global minimum energy, regardless of the initial configuration.

This fact is associated with the complexity of the system, composed of several PSS macromolecules confined between two rGO sheets, in which total relaxation of the system may require simulation times of macroscopic order and, therefore, inaccessible to MD simulations. Although there are techniques to accelerate equilibration, none of them effectively guarantee complete relaxation of the system, which may require several hundred nanoseconds [15,18,19]. This can be associated with the slow dynamics of the PSS polymer due to the interactions with rGO that reduce the evolution of the system to the global minimum energy state.

Therefore, even though the energy remained stable during the simulations, the final structures obtained may still evolve into more energetically favorable conformations if much longer simulation times are employed. A strategy to overcome this limitation consists of performing a large number of simulations starting from different randomly generated initial configurations. This would increase the statistical representation of simulations, reducing the bias of specific initial configurations and, consequently, increasing the reliability of the results. Therefore, in this manuscript we present the results obtained from the average over ten distinct MD simulations. From this, it is possible to identify a trend in the behavior of PSS on the rGO sheet depending on the sulfonation fraction. Furthermore, conducting a case study allows the identification of how the conformation of the film directly affects the dynamic properties of the solvent and elucidates the mechanism underlying the selectivity.

### 2.1. Structural Analysis of rGO/PSS

#### 2.1.1. Global Structure

**Figure 1:**
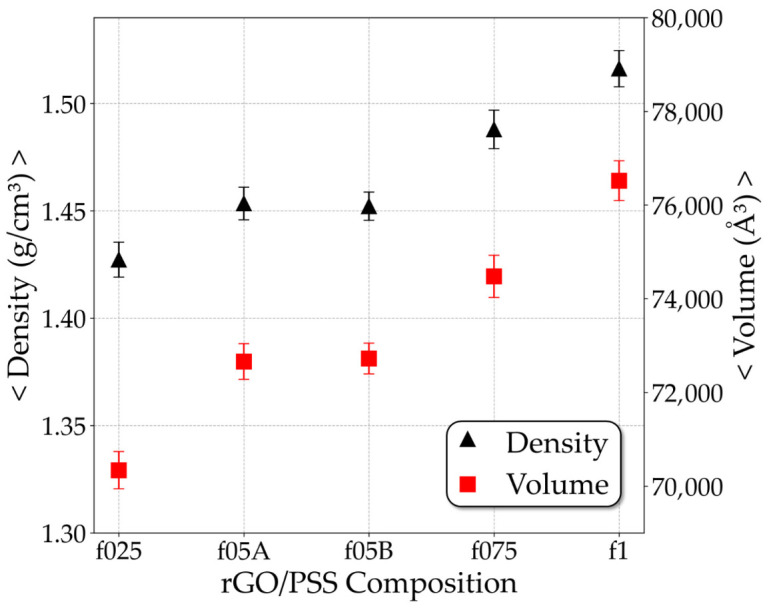
These figures show the average mass density (left axis) and volume (right axis) for each rGO/PSS film composition.

Figure 1 shows the average of the mass density and the volume for each relaxed rGO/PSS composition. Both density and volume simultaneously increase as the sulfonation fraction of PSS increases. This increase in density can be associated with the greater mass present in larger fractions systems, due to the $SO_{3}^{-}$ groups and the hydronium counterions responsible for their neutralization. On the other hand, the simultaneous increase in density and volume observed for different fractions cannot be explained solely by the increase in mass. The volume increase is primarily driven by the expansion of film thickness (Figure S2), which is probably related to the dispersion of the polymer chains between the rGO sheets. Thus, the first analysis on the polymer conformation was done by measuring the gyration radius ($R_{g}$) of the PSS, which indicates the degree of dispersion of the polymer chain around its center of mass. Since it is an extensive property, in the context of MD simulation, these values are meaningful only when compared among the different rGO/PSS films. For comparison purposes, we simulated the PSS free in solution to obtain the reference $R_{g}$ values.

**Figure 2:**
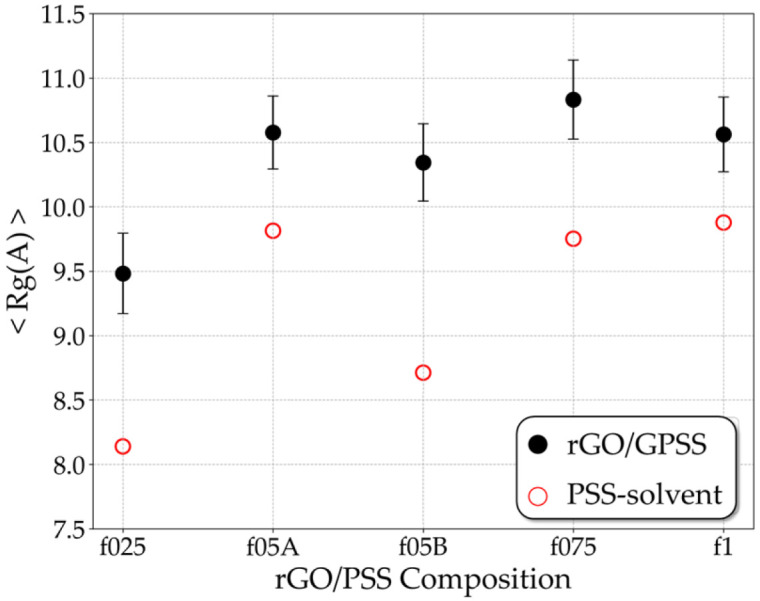
Gyration radius ($R_{g}$) of PSS freely dispersed in solvent represented by open red circles and PSS embedded in rGO/PSS films represented by full black circles for different sulfonation fractions (f025, f05A, f05B, f075 and f1).

Figure 2 shows the average $R_{g}$ for the five types of PSS in solution (~39 wt% solvent) and under confinement. In solution, the $R_{g}$ increases slightly with the sulfonation fraction, except for the f05A configuration. This trend might be associated with the chain uncoiling caused by the strong electrostatic repulsion between adjacent $SO_{3}^{-}$ groups [13]. Under confinement between rGO sheets, however, no systematic dependence on the sulfonation fraction is observed. In all cases, the confined PSS exhibits a larger $R_{g}$ than in solution, indicating that interactions with the rGO surfaces promote chain extension regardless of the sulfonation fraction.

**Figure 3:**
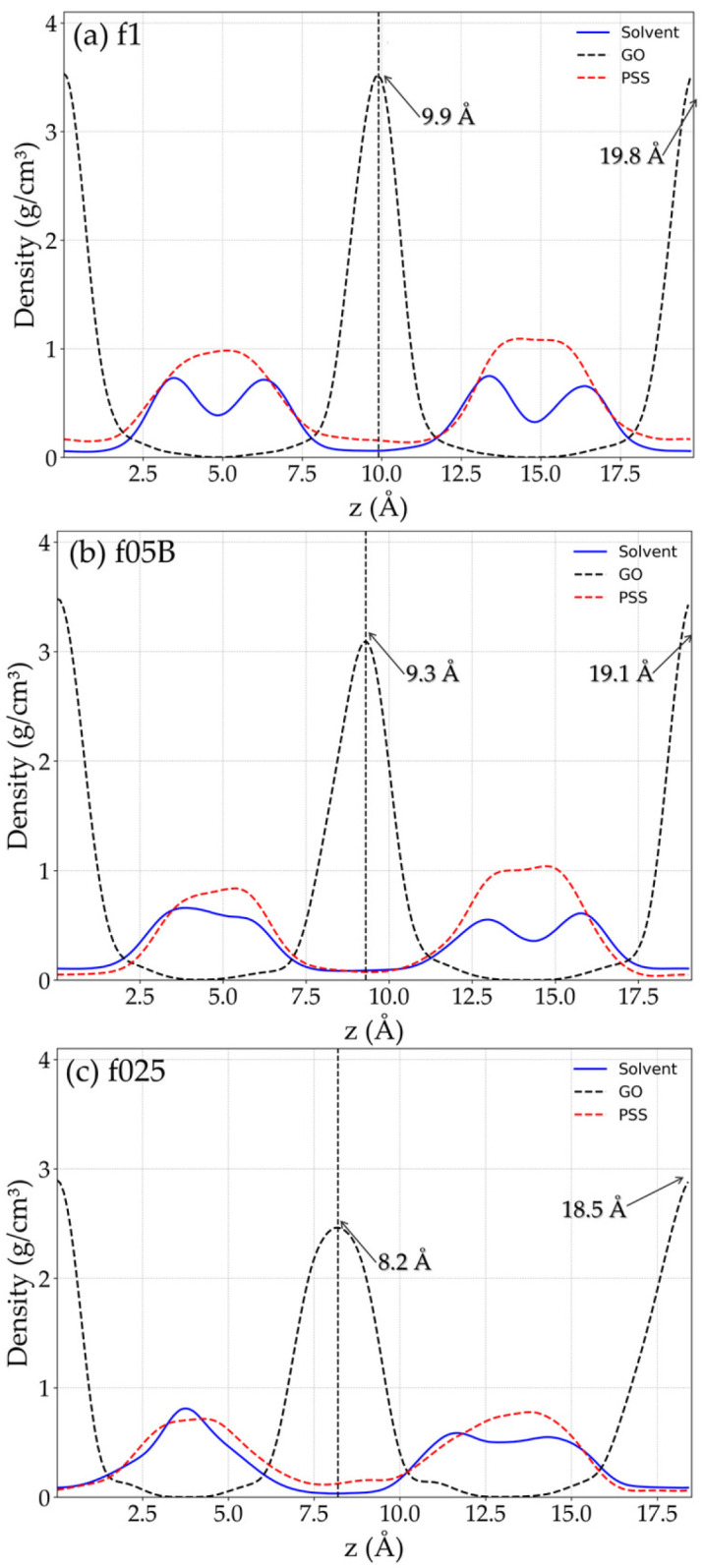
The mass profiles of rGO (black), polymer (red) and solvent (blue) for films with sulfonate fraction (**a**) f1 and (**b**) f05B and (**c**) f025, representing the typical distribution of the components along the z-direction.

To further analyze the polymer conformation in confinement, as well as the distribution of the rGO/PSS parts along the direction perpendicular to the rGO sheets, the mass density profile of each component was calculated along the z-axis, as shown in Figure 3. We have chosen to present the most representative cases, namely the most hydrophilic (f1), the intermediate (f05B) and the most hydrophobic PSS (f025). The complete results are provided in SI, together with additional equilibration and convergence evidence for the studied cases (Figure S8). The graphs clearly show the position of rGO sheets, represented by the two well-defined peaks of the rGO density profile (black dashed lines). For clarity and to avoid misinterpretation arising from the periodicity in the z-direction, all curves were shifted so that the first rGO peak is positioned at 0. The peak widths reflect the sheet rugosity, while their separation indicates the typical interlayer distance. Meanwhile, the PSS and solvent density profile in red and blue lines, respectively, indicate the polymeric regions between the sheets. Thus, it is possible to identify two polymeric regions: Region 1 (first half) and Region 2 (second half). The density profiles reveal a clear distinction between the most hydrophilic PSS configuration (f1) and the most hydrophobic one (f025). In the f1 case, the solvent is more structurally organized (blue lines), with the formation of double solvent layers. Indeed, the presence of sulfonate groups enhances surface wettability, promoting the spreading of water and stabilizing interfacial layering. In contrast, the hydrophobic configuration shows broader rGO peaks and no solvent layering effect. Under these conditions, water is likely more confined between the sheets and does not spread uniformly along the rGO surface.

Moreover, the rGO interlayer distance is affected by the distribution of the polymer. In the f1 configuration, Region 1 and Region 2 both have a thickness of 9.9 Å with similar solvent and polymer density profiles. These results indicate that the f1 film exhibits a largely uniform internal structure, with symmetric regions of equal thickness, equivalent polymer distribution, and the formation of solvent bilayers in both regions. In contrast, the f025 configuration, the Region 1 thickness dropped to 8.2 Å, while Region 2 increased to 10.3 Å. This occurred because the polymer has partially passed from Region 1 to Region 2, unbalancing the distribution of the polymer chains in the film. Although the passage of the polymer from one region to another may be an artifact of the very short chain length used in this model, the phenomenon likely reflects the underlying balance of interactions between rGO–PSS and rGO–rGO.

**Figure 4:**
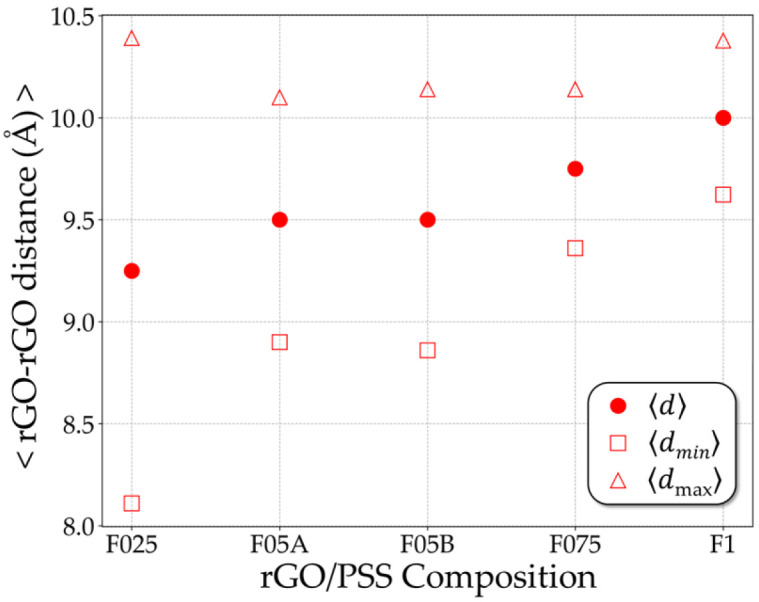
Average interlayer distances between rGO sheets, 〈d〉. The shortest, $\langle d_{min}\rangle ,\;$ and the largest, $\langle d_{max}\rangle$, interlayer separations are represented by square and triangle open symbols, respectively.

These observations are to a certain extent reproduced in other simulations of f1 and f025 configurations. The intermediate case, as observed for the f05 configuration, displays intermediate behaviors, with formation of bilayer less structured and intermediate symmetries. To generalize the results so far, Figure 4 shows the average interlayer distances between rGO sheets (see Figure S9 for complete data). Due to the periodic boundary conditions of the simulation box, two characteristic distances are identified: the shortest and the largest interlayer separations. It is possible to trace a behavioral trend, in which the shortest rGO–rGO distance increases with the sulfonation fraction. This behavior indicates that higher sulfonation promotes a more homogeneous distribution of the polymer within the film, effectively preventing close restacking of the rGO sheets. Conversely, when the $SO_{3}^{-}$ content is reduced, the polymer becomes less uniformly distributed, allowing the rGO sheets to approach each other. This effect arises from the balance among rGO–rGO attractive interactions, PSS–rGO interactions and PSS–PSS repulsive interaction, suggesting that the rGO–rGO interactions become more dominant at lower sulfonation degrees.

Indeed, as reported by Maddalena et al. [3], the PSS acts as a multifaceted agent in stabilizing rGO dispersions and, thus, avoiding rGO stacking. Mechanistically, the sulfonate groups of PSS impart a strong negative charge to the composite, producing electrostatic repulsion that counteracts van der Waals-driven restacking and aggregation of rGO sheets. Our findings are qualitatively in agreement with these studies, since the reduction of $SO_{3}^{-}$ mediated repulsive interactions favor rGO–rGO attraction. To further detail the balance of interactions among the film components, the next section explores the nature of these interactions and their role in the orientation of the polymer chains on the graphene surface.

#### 2.1.2. rGO–PSS Interaction

**Figure 5:**
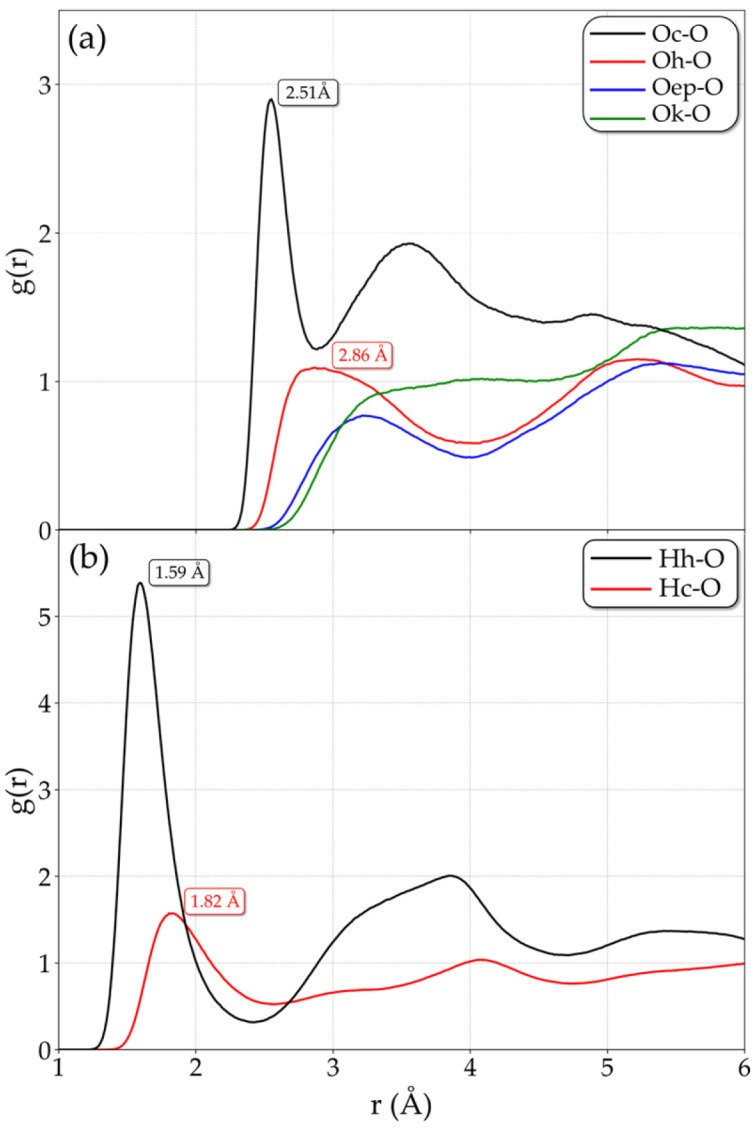
(**a**) RDFs between oxygen of the $SO_{3}^{-}$ group (O) and the oxygen of rGO functional groups, such as carboxyl (Oc), hydroxyl (Oh), epoxy (Oe) and carbonyl (Ok). (**b**) RDFs between oxygen of the $SO_{3}^{-}$ group (O) and hydrogen of the functional groups carboxyl (Hc) and hydroxyl (Hh) with the peaks at 1.59 and 1.82 Å, respectively, highlight that the interaction rGO–PSS occurs via H-bond formation with these two groups.

**Figure 6:**
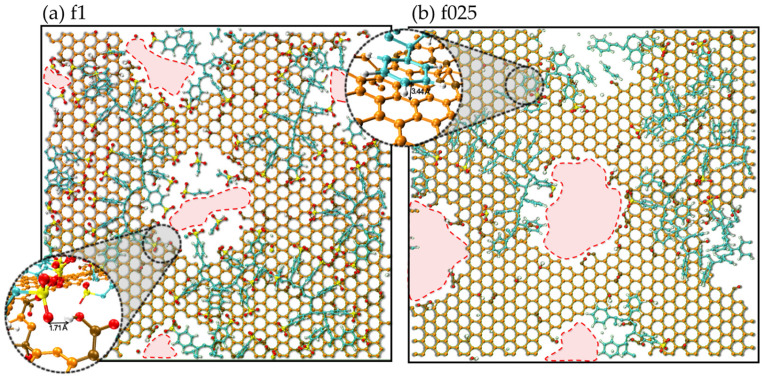
(**a**) Snapshot of the f1 configuration showing the polymer chain (cyan beads) adjacent to the rGO pores (holes). The red dashed lines highlight the reduction in pore aperture due to polymer obstruction. The indicated distance between the $SO_{3}^{-}$ (yellow and red beads) and –COOH groups (ochre, red and white beads) illustrates the hydrogen bond formation. (**b**) Snapshot of the f025 configuration showing unobstructed pores (red dashed lines). The alignment of the benzene ring (cyan beads) with the hexagonal rGO network (orange beads) suggests π–π interaction.

Understanding the interaction between PSS and rGO requires detailed interfacial characterization. At the local scale, this can be assessed by analyzing the shortest interaction distances between PSS and rGO atoms. This distance can be obtained from the position of the first peak in the radial distribution function (RDF), which reflects the most probable distance between atomic pairs. Thus, Figure 5a shows the RDF between the oxygens (O) of the PSS sulfonate group with those of the rGO functional groups: carboxyl (Oc), hydroxyl (Oh), carbonyl (Ok) and epoxy (Oep). Prominent peaks located at 2.5 and 3.0 Å are observed in Oc–O and Oh–O RDFs, which means that the $SO_{3}^{-}$ first interacts with carboxyl (-COOH) the hydroxyl (-OH) groups. This interaction occurs via the formation of hydrogen bonds between the oxygen of $SO_{3}^{-}$ and the hydrogen atoms of the functional groups, indicated by the RDF peaks at 1.59 and 1.82 Å displayed in Figure 5b and illustrated in Figure 6a. This primary interaction of $SO_{3}^{-}$ with rGO via hydrogen bonds is observed in all different films, as can be verified in Figure S10. For the other functional groups, the distance between interactions is greater and the peaks are not as well defined, suggesting secondary roles in the interfacial property between PSS and rGO.

Another type of interaction between PSS and rGO emerges as the sulfonation fraction decreases, becoming evident already at f075. The phenyl rings of PSS start to interact with the sp^2^ carbons of the rGO network through the π–π stacking interaction. These interactions occur at typical distances between 3.4 and 3.6 Å. Although it is an interaction of a quantum nature, not explicitly represented in MD simulations, its occurrence can be inferred by the proximity and alignment of the rings with the graphene sp^2^ network, as shown in Figure 6b. Beyond π–π interactions, the hydrophobic segment of the PSS chain interacts with the hydrophobic domains of rGO via van der Waals forces. Both types of non-covalent interactions are commonly observed in graphene-based composites containing polymers with aromatic rings or hydrophobic polymers [1,20].

Since PSS is a polyelectrolyte containing both hydrophobic and hydrophilic components, it can interact with rGO sheets through two distinct mechanisms: (i) hydrophobic interactions, driven by π–π stacking and van der Waals forces, and (ii) hydrophilic interactions mediated by its sulfonate groups [21,22]. These interaction mechanisms are consistent with previous studies on aromatic polymers and surfactants adsorbed on graphene-like surfaces, where π–π interactions are widely considered a key stabilizing factor [2,20]. Although direct experimental evidence of sulfonate-group-mediated hydrogen bonding in PSS–rGO composites remains scarce, ionic interactions between functionalized polymers and graphene-based materials are widely reported, and hydrogen bonding is known to facilitate adsorption and dispersion in aqueous media [23,24].

Our simulations corroborate these mechanisms reported in the literature. Specifically, we observe hydrogen bond formation between rGO functional groups and the sulfonate moieties of PSS, characterizing hydrophilic interactions. In parallel, π–π stacking between PSS phenyl rings and the sp^2^ domains of rGO evidences hydrophobic interactions. However, analysis of the simulation trajectories indicates that, due to steric constraints and intramolecular polymer interactions, the alignment of the benzene rings with the sp^2^ network is maintained for shorter periods compared to the hydrogen bonds. This suggests that, in the present system, hydrophilic interactions play a more dominant role than hydrophobic ones in the overall PSS–rGO interaction.

These results demonstrate the dual interaction nature of PSS at the rGO interface. The balance between these driving forces determines the conformation of PSS on the rGO surface and, consequently, the resulting interfacial structure. Moreover, different sulfonation fractions lead to distinct interaction balance. For instance, in the f1 configuration, the chains tend to approach and even occupy the rGO pores due to the strong interaction between the groups $SO_{3}^{-}$ and −*COOH* located at the pore edges. This behavior is supported by the $SO_{3}^{-}$ density distribution shown in Figure S11a, where sulfonate groups are found at positions corresponding to the rGO sheets. Their presence in these regions indicates that the $SO_{3}^{-}$ groups are adjacent to the pores (holes) in the rGO sheets. As a consequence, the pore aperture is significantly reduced, as illustrated in Figure 6a, due to polymer blockage. This reduction is expected to strongly influence solvent transport through the film, as will be detailed later.

In contrast, the low amount of $SO_{3}^{-}$ in the f025 configuration leads to a reduction in the interaction between the polymer chain and the rGO sheet functional groups. This weaker interaction together with the *π*–*π* interactions favors the dispersion of the polymer in regions of the rGO surface that do not present defects or functional groups. Consequently, the pores are unobstructed, as illustrated in Figure 6b. Moreover, the overall rGO–PSS interaction is weakened as the $SO_{3}^{-}$ content decreases. This may eventually facilitate the passage of the polymer through the defects and, consequently, promote closer rGO–rGO interactions, as discussed in the previous section. The dispersion of the polymer chains, together with the rGO–rGO interlayer distances, plays a key role in governing solvent transport through the film, as discussed in the following section.

### 2.2. Water and Methanol Selective Diffusion

**Figure 7:**
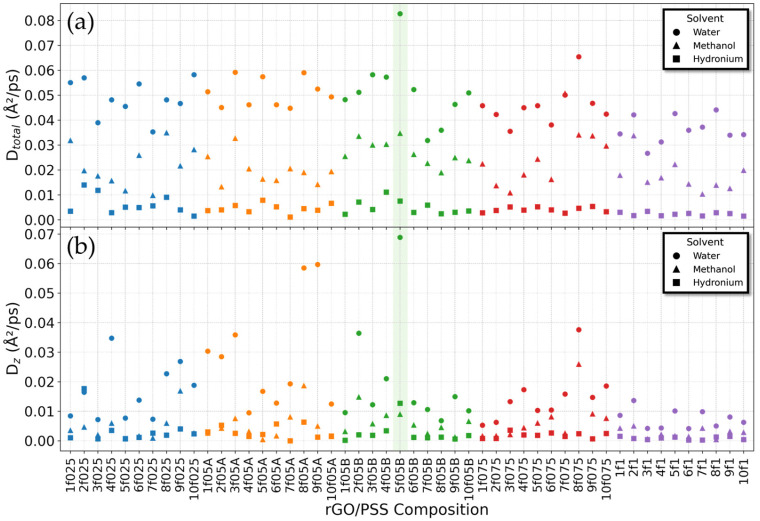
(**a**) Total diffusion coefficients and (**b**) z-component diffusion coefficients of water (circles), methanol (triangles), and hydronium (squares). The 5f05B configuration is highlighted in green shading to emphasize that the water-to-methanol diffusion ratio rises from 2.5 (total diffusion) to 7.7 when considering diffusion along the z-direction.

To elucidate how the structural conformation of the film influences solvent dynamics, the mean square displacement (MSD) and the associated diffusion coefficient were analyzed. Comparing the diffusion behavior of each solvent component is particularly important, as experimental evidence indicates that rGO/PSS acts as the primary barrier to methanol permeation through the PAH–rGO/PSS polymeric film, thereby preventing poisoning of the polyelectrolyte in DFMC cells [10]. Solvent diffusion occurs through permeation across the film layers, where the rGO/PSS simultaneously acts as a physical barrier to methanol transport while allowing water permeation through its stacked structure. In this work, permeation across the film necessarily occurs along the z-direction, through the pores of the rGO sheets. Figure 7 presents both the global and z-component diffusion coefficients of the solvent species—water, methanol, and hydronium—for all rGO/PSS configurations. For each configuration, diffusion coefficients were extracted from a linear fit of the MSD between 500 and 1500 ps of the production phase, considering only the linear regime associated with diffusive behavior. In all cases, the fittings yielded a coefficient of determination *R*^2^ > 0.9.

Notably, diffusion along the z-axis does not necessarily mirror the global diffusion trends shown in Figure 7a. This distinction is especially clear when comparing water and methanol: in the z-direction, their diffusivities differ even more markedly than in the overall diffusion. For instance, in configuration 5f05B, the global diffusion coefficient of water is approximately 2.5 times higher than that of methanol (*D*water = 0.082 Å^2^/ps; *D*methanol = 0.034 Å^2^/ps). Although this ratio exceeds that obtained for the isolated PSS–f05B chain in solution (*D*water = 0.18 Å^2^/ps; *D*methanol = 0.09 Å^2^/ps), it is not sufficient to indicate an effective separation mechanism. In contrast, the z-direction diffusion exhibits a much larger disparity, with water diffusing approximately 7.7 times faster than methanol (*D*water = 0.069 Å^2^/ps; *D*methanol = 0.0089 Å^2^/ps). The selective metric (*D*water/*D*methanol) for all configurations is presented in Figure S12.

**Figure 8:**
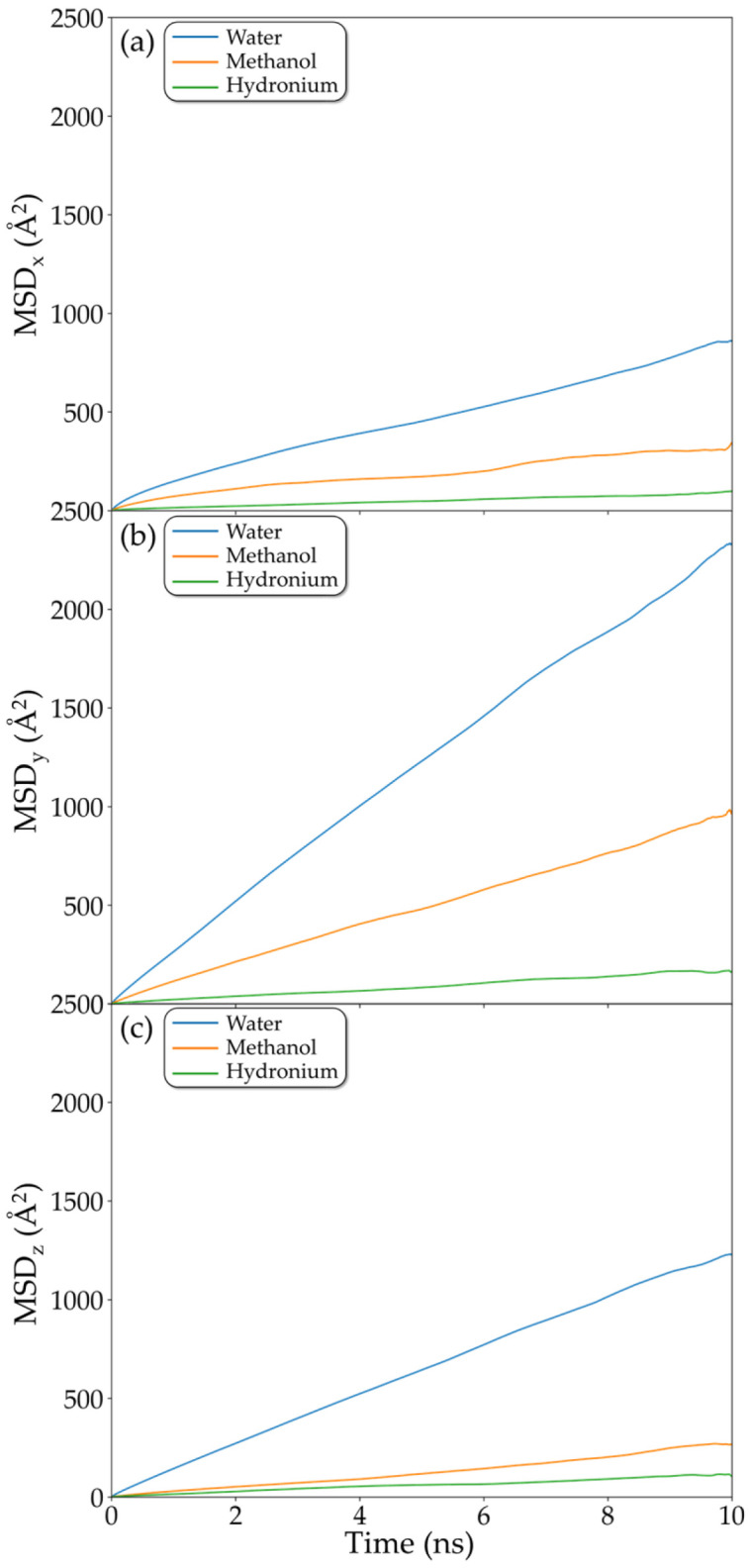
Mean squared displacement of water, methanol and hydronium along the (**a**) x-, (**b**) y-, and (**c**) z-directions.

**Figure 9:**
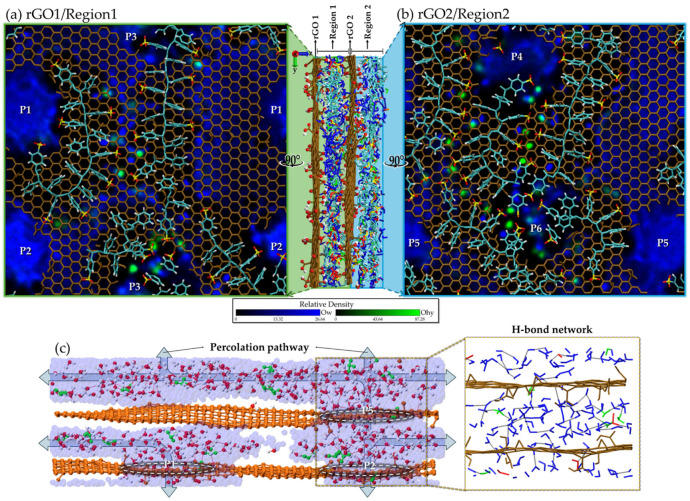
(**a**,**b**) Snapshot of rGO (carbon in ochre) and PSS chain (carbon in cyan, oxygen in red and sulfur in yellow) with heat maps of water (blue) and hydronium (green) superimposed. The rGO pores are labeled P1, P2 and P3 (rGO1/Region 1) and P4, P5 and P6 (rGO2/Region 2). The figure illustrates a polymeric barrier oriented along the Y-direction in Region 1 and the unobstructed pores P1, P2 and P5, as discussed in the text. (**c**) Snapshot of the lateral view showing the alignment of pores P2 and P5, illustrating the percolation pathway that results in a connected water cluster and a hydrogen-bond network across the film.

Given that the primary aim of this study is to evaluate water–methanol transport and separation along the z-direction, we conducted a detailed analysis of the best case, the 5f05B configuration, which displayed the highest water diffusivity and highest discrepancy relative to methanol, in addition to significant hydronium diffusion. To ensure reliability, case 5f05B was subjected to an extended thermalization period, followed by a long production run, resulting in a total simulation time exceeding 50 ns. Figure 8 shows the directional MSDs of water, methanol and hydronium. Figure 8c highlights the difference in the slopes of water and methanol MSD curves, confirming the superior mobility of water, while methanol diffusion remains strongly hindered in this direction. More notably, however, anisotropic behavior is detected within the XY plane. In the X-direction (Figure 8a), both water and methanol display substantially reduced diffusion relative to the Y-direction (Figure 8b). This in-plane anisotropy can be rationalized by inspecting the spatial organization of the polymers adsorbed on the rGO surfaces. The diminished diffusion along the X-direction results from the polymeric barrier oriented along the Y-direction, as illustrated by the polymer snapshots shown in Figure 9. Although the observed anisotropy is noteworthy, these results cannot be generalized, as the effect is not reproducible in other f05B configurations. Because the polymer arrangement is governed primarily by its interactions with the rGO functional groups and by π–π interactions—and because these functional groups are randomly distributed on the rGO surface—there is no preferential orientation that the polymer consistently adopts.

Now, to further elucidate the differences in water and methanol diffusive behavior, it is necessary to analyze both the global confinement effects and the local heterogeneities in the interactions between the solvent and the film components. As a first step toward understanding these local interactions, we calculated RDFs between the solvent species, i.e., water (Ow) and methanol (Om) oxygen atoms, and the functional groups, i.e., hydroxyl (Ohi), carboxyl (Oc1) and sulfonate (O) oxygen atoms, as shown in Figure S13. It is observed that the interaction distances of the functionals with water ($r_{OwOhi}$ = 2.86 Å, $r_{OwOc1}$ = 2.64 Å and $r_{OwO}$ = 2.67 Å) and methanol ($r_{OmOhi}$ = 2.86 Å, $r_{OmOc1}$ = 2.63 Å and $r_{OmO}$ = 2.62 Å) are compatible with the formation of hydrogen bonds, both with the functional groups of the rGO and the sulfonated groups of PSS. This result indicates that there is no preferential interaction of rGO/PSS film with methanol or water that justifies the restriction on methanol diffusion. Both interact through hydrogen bonds with functional groups and sulfonates.

After examining the local interaction patterns through the RDF analysis, we next address the influence of global confinement on the solvent distribution. To investigate these confinement effects, heat maps were computed in the XY plane to represent the time-average spatial density of the solvent components throughout the simulation. These maps are expressed in terms of relative density, $\rho_{rel}={\langle \rho \left(x,y\right)\rangle }_{t}/\rho_{bulk}$, which allows the identification of solvent accumulation ($\rho_{rel}>1$) and depletion ($\rho_{rel}<1$) induced by confinement. For this analysis, the film was divided into two regions across its thickness: Region 1 (first half) and Region 2 (second half). Figure 9a and Figure 9b show the corresponding heat maps and polymer configurations for Regions 1 and 2, respectively. These maps reveal the spatial regions mostly occupied by each species during the 10 ns of production simulation, thereby highlighting how confinement modifies the local solvent distribution compared to the bulk environment.

For instance, the hydronium heat maps (green color) indicate strong confinement of hydronium ions located in bright green areas near the PSS side chains functionalized with a $SO_{3}^{-}$ group. The hydronium remains confined within the hydrophilic corridors formed between these side chains, a pattern observed in both Region 1 and Region 2. This confinement is further supported by the RDFs between the hydronium oxygen (Ohy) with the functional groups of rGO (hydroxyl (Ohi) and carboxyl (Oc1)) and sulfonate (oxygen (O)), shown in Figure S14. The interaction *O* − *Ohy* represents the shortest distance ($r_{OOhy}$ = 2.49 Å) compared to the other interactions ($r_{OhiOhy}$ = 2.92 Å and $r_{Oc1Ohy}$ = 2.86 Å). This indicates a strong electrostatic interaction between the counterion $H_{3}O^{+}$ and the ionic group $SO_{3}^{-}$, resulting in hydronium trapping and consequently low diffusion. The stronger affinity for the sulfonate oxygen is further supported by the coordination numbers obtained from integrating the RDF up to r = 3.5 Å: $N_{OOhy}$ = 0.54, $N_{Oc1Ohy}$ = 0.17 and $N_{OhiOhy}$ = 0.17. These values represent the average number of *Ohy* surrounding each functional group and confirm the higher hydronium concentration near $SO_{3}^{-}$ compared to the rGO groups.

From the water heat maps (blue color) in Figure 9, we can clearly identify a higher concentration of water on the left side of Region 1 and on the right side of Region 2, corresponding to areas without polymer chains. This accumulation forms a continuous percolation pathway in both the y- and z-directions, resulting in a connected water cluster as illustrated in Figure 9c. Such a pathway is essential for enhancing water diffusion and promoting the formation of a hydrogen bond network across the film, which ultimately could create favorable conditions for proton conduction. This feature is not consistently observed in other rGO–PSS configurations. The formation of this pathway is associated with two key factors. First, the rGO pores are not obstructed by the polymer chain, as observed for pores P1, P2 and P5. Second, a partial or complete alignment of the pores from distinct rGO sheets occurs, as exemplified by the alignment between pores P2 and P5. For comparison with other rGO–PSS configurations, this pore alignment is primarily responsible for the significantly higher diffusion observed in the 5f05B configuration relative to the others. In addition, unobstructed pores are crucial for water diffusion. As a counterexample, the 4f1 film discussed in Figure 6a presents pores filled with PSS, which drastically reduces the diffusion coefficient in z (Figure 7).

**Figure 10:**
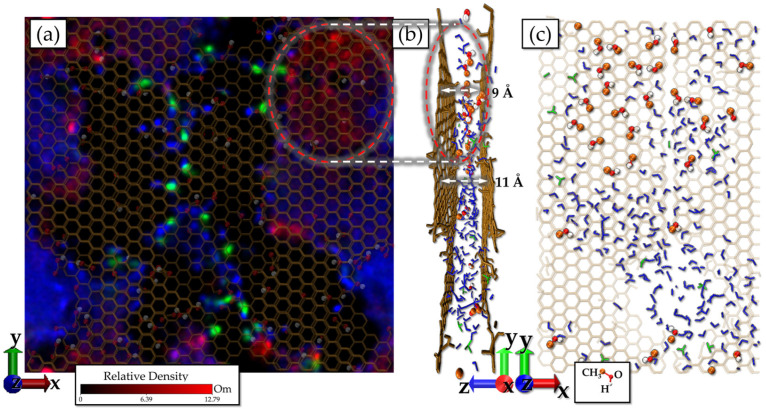
(**a**) Heat map of methanol (red color), water (blue color) and hydronium (green color) showing the accumulation of the methanol at the upper right and left area. (**b**) Snapshot of the lateral view showing trapped methanol forming a monolayer within the narrowed rGO–rGO channel. (**c**) Snapshot illustrating the preferential orientation of methanol molecules, with the C-O-H plane parallel to the rGO sheet.

The obstruction to the transport of methanol across the rGO/PSS is finally explained by the methanol heat map (red color) obtained over the 2 ns simulation, as shown in Figure 10. Note the presence of regions with a higher concentration of methanol, characterized by red spots on the map. The most pronounced region appears in the upper right area, highlighted with a red circle, indicating the confinement of the *CH*_3_*OH* molecules. It is observed that this confinement occurs in areas where the rGO sheets are primarily composed of the sp^2^ network and there is no presence of polymers. Another relevant factor is the distance between the rGO sheets. The methanol entrapment mechanism is mainly associated with the formation of a pure methanol monolayer in regions where the rGO sheets are narrowed, at around 9 Å, as indicated in Figure 10b. In these confined regions, methanol preferentially adopts an orientation in which the C–O–H plane is parallel to the rGO sheets, as shown in Figure 10c. This orientation maximizes the van der Waals interactions with both surfaces, thus promoting molecular trap. In contrast, in wider regions (~11 Å), water bilayers are formed, within which methanol becomes diluted, leading to reduced confinement and less restriction to its diffusion.

Overall, the results show that although there are no explicit structural barriers to the diffusion of methanol or hydronium through the defects aligned in the rGO/PSS 5f05B configuration, there is a lower diffusion of these molecules along the *Z*-axis due to the trapping of hydronium in the hydrophilic corridors between the PSS chains, resulting from the electrostatic interaction and the confinement of methanol between the rGO sheets due to the formation of narrow 2D channels (~9 Å). The underlying barrier mechanism is reproducible across all f05B configurations; however, the quantitative results for the 5f05B case are not generalizable due to the intrinsic limitations of our model. Because these results are highly sensitive to the rGO pore size and alignment, our findings are intended to elucidate the mechanisms underlying rGO/PSS barrier properties and identify specific structural characteristic correlation, rather than providing quantitatively precise predictions.

In a more general context, it is important to note that the formation of 2D narrow channels in the composite films depends on the concentration of PSS and rGO, requiring the rGO to be enveloped by the polymer, which prevents the rGO sheets from stacking. Excess polymer would increase the distance between the sheets, which would prevent the formation of optimal 2D channels with the thickness required to trap methanol. The scarcity of PSS in the film would lead to the stacking of the rGO sheets, making it difficult for the solvent to diffuse. In this work, the same concentrations (~40 wt% of PSS) used in the synthesis of the experimental study were used [2]. This concentration, together with the intermediate sulfonation fraction, was found to provide the optimal balance between structural features with the selective diffusion of methanol through the rGO/PSS.

## 3. Computational Methods

**PSS model:** Polystyrene sulfonate (PSS) is an anionic polyelectrolyte composed of a hydrocarbon main chain (backbone) and a side chain containing an aromatic ring functionalized with a sulfonate group ($SO_{3}^{-}$) (Figure S15). The degree of polymerization corresponds to the number of monomers present in the chain, while the sulfonation fraction is defined as the ratio between the number of side chains containing the ionizable $SO_{3}^{-}$ group and the total number of side chains [13]. In this work, PSS chains with 16 monomers and an end-to-end length of 47.44 Å were used. The polymer size was chosen to match the dimensions of the simulated rGO sheet. Using multiple short chains was preferred over a single extended chain, as very long polymers would lead to impractically long relaxation times in atomistic simulations. Four PSS types were generated: a fully sulfonated configuration (*f* = 1) and partially sulfonated configurations with *f* = 0.75, *f* = 0.5, and *f* = 0.25. For *f* = 0.5, two variants were considered: *f*05A, in which all sulfonate groups on one side of the chain were removed, and *f*05B, in which they were removed alternately (Figure S16). Hydronium counterions (H_3_O^+^) were added to fully neutralize the system.

The representation of PSS was carried out using an atomistic model for the atoms of the side chains, while the main chain was represented through a united-atom approach, which consists of treating the hydrocarbons in the backbone (CH, CH_2_, and CH_3_) as neutral pseudo-atoms with their respective atomic masses. Table S1 presents the partial charges and the LJ parameters used for interactions between identical particles. The Lorentz–Berthelot rule [25] was employed to determine the cross parameters. Tables S2–S5 contain the equations and parameters of the bonded potentials. The classical force field was adapted from Miyazaki et al. [10] based on the model originally proposed by Carrillo and Dobrynin (2010) [13].

**rGO model:** Reduced graphene oxide (rGO) consists of a graphene basal plane functionalized with oxygen-containing functional groups, namely hydroxyl (−OH), epoxy (−O−), carbonyl (−CO), and carboxyl (−CO_2_H). Hydroxyl and epoxy groups are generally distributed across the basal plane, while carbonyl and carboxyl groups are predominantly located at the sheet edges [26]. The primary distinction between rGO and graphene oxide (GO) is their oxidation level, which is significantly decreased during the reduction process. This reduction can be achieved through various methods, including chemical routes [27] and thermal treatments such as high-temperature annealing [28]. The reduction of GO often induces structural defects, such as vacancies and lattice distortions in the carbon network [29]. The degree of oxidation of a GO sheet is typically expressed by the oxygen-to-carbon ratio (O/C), although there is no universal consensus on the exact range that distinguishes GO from rGO [30].

To computationally build the rGO sheet, an in-house C program [31] was used to generate different configurations of rGO from a pristine graphene sheet. The code allows for control over the oxidation level of the sheet, the types of oxygenated functional groups to be added, and the inclusion of topological hole-type defects. Hydroxyl and epoxy groups are distributed across the sheet in an almost random manner, following the restrictions of not adding two functional groups to the same carbon atom and not placing two hydroxyls on neighboring atoms. Carbonyl and carboxyl groups are attached to edge carbon atoms, either at the sheet boundary or at pore sites.

For this work, an rGO sheet with an overall oxidation degree of 7% was generated, comprising 2% epoxy groups, 2% hydroxyl groups, 2% carboxyl groups, and 1% carbonyl groups, reproducing the composition reported in the experimental study of PSS-embedded rGO [10]. Periodic boundary conditions (PBCs) were applied to mimic an infinite sheet. To enable solvent and polymer permeation through the film, three topological defects (holes) were introduced, each removing approximately 15% of the atoms in the corresponding region (Figure S17). The use of relatively large pores, rather than explicitly modeling a finite rGO sheet, helps avoid artifacts that arise from simulating small graphene-based surfaces under PBC. The resulting rGO sheet has dimensions of 62.4 × 60.2 Å and contains a total of 1302 atoms. The classical force field used for the rGO was based on the model originally proposed by Jiao and Xu (2015) [32]. The charge and Lennard-Jones (LJ) parameters are presented in Table S6, and the bonded potential parameters are provided in Tables S7–S9.

**Simulation details:** Molecular Dynamics (MD) simulations of the rGO/PSS + solvent composite were carried out. The number of PSS chains in the simulation was determined based on the mass percentage ratio between rGO and PSS reported in reference [9,10], which is approximately 60% rGO and 40% PSS. For the calculation, the PSS *f*1 model was used, yielding a mass fraction of 56.91% rGO and 43.09% PSS. The solvent consisted of 90% water and 10% methanol, corresponding to a solvent content of approximately 18 wt% relative to the total film mass. Thus, the initial configurations were constructed using Packmol software (Version 20.3.1) [33] by adding two parallel rGO sheets in the *xy* plane, separated by 50 Å, and four PSS chains were inserted at random positions, with two placed on each side of the rGO sheets. A total of 600 water molecules, 60 methanol molecules, and the amount of hydronium ions required to neutralize the PSS charge were then randomly distributed in the simulation box (Figure S18). Ten distinct initial configurations were prepared for each PSS sulfonation fraction, totaling 50 initial distinct simulations.

The simulations were performed using the LAMMPS software (Large-scale Atomic/Molecular Massively Parallel Simulator—Version 29Oct2020) [34], employing the Nosé–Hoover thermostat and barostat [35] with relaxation times of 100 and 1000 times the timestep, respectively. The timestep used in the simulations was 1 fs, and the pressure was set to ambient conditions (1 atm). The force fields used were those previously described for PSS and rGO; for water molecules, the SPC/E model [36] was chosen, and for methanol, the TraPPE model [37] was used. Water molecules were treated as rigid using the SHAKE algorithm [38]. A cutoff radius of 15 Å was applied for both LJ and Coulomb interactions. Long-range electrostatic interactions were handled using the PPPM method [39]. Periodic boundary conditions (PBC) were employed in all directions to simulate a bulk-like environment.

The simulations began with energy minimization using conjugate gradient algorithms to relax atomic positions and avoid unphysical initial configurations. Thermalization started by gradually reducing the box height of 20 Å under the canonical ensemble (NVT) over a period of 200 ps, followed by an additional 200 ps at T = 1500 K in the same ensemble. Because the force field does not permit bond breaking, this high initial temperature does not damage the film components, but it serves as a strategy to accelerate equilibration. The system was then cooled from 1500 K to 300 K over 200 ps in the isotropic isothermal–isobaric ensemble (NPT) and held at 300 K for another 200 ps. Subsequently, an anisotropic NPT simulation at 300 K was performed for 4.2 ns, completing a total of 5 ns of thermalization. For the production stage, the simulation box edges were fixed to the average value obtained from the last 2 ns of equilibration. The system was equilibrated for 100 ps at 300 K in the NVT ensemble, followed by an additional 100 ps for stabilization. Afterward, the system was simulated for 2 ns in the canonical ensemble (NVT) at ambient temperature for data collection.

## 4. Conclusions

Classical MD simulations were employed to explore the structural and dynamic properties of rGO/PSS thin films at different polymer ionization fractions in a solvent mixture of water, methanol, and hydronium ions. These studies examine the balance between hydrophobic and hydrophilic interactions at the rGO–PSS interface and reveal that hydrophilic interactions play a more dominant role than hydrophobic ones in the overall PSS–rGO interaction. Consequently, the rGO–PSS interaction is weakened as the $SO_{3}^{-}$ content decreases, favoring stronger rGO–rGO attraction and consequently reducing the interlayer distances. The dispersion of the polymer chains, together with the rGO interlayer spacing, plays a key role in governing solvent transport through the film. The water transport across the film is enhanced when a connected water cluster percolates through it. This percolative pathway is characterized by the formation of a hydrogen bond network across the film that could favor proton mobility. At last, the most significant finding of these studies concerns the mechanism underlying water–methanol selectivity. Methanol entrapment is mainly associated with the formation of narrow 2D interlayer channels, within which a methanol monolayer is confined through maximized van der Waals interactions with both sheets. The ideal scenario for methanol confinement requires optimal interlayer spacing, which is governed by a proper balance between the PSS and rGO concentrations, as well as the degree of sulfonation.

## Data Availability

The original contributions presented in this study are included in the article and Supplementary Materials. Further inquiries can be directed to the corresponding author.

## Supplementary Materials

The following supporting information can be downloaded at https://www.mdpi.com/article/10.3390/molecules31101657/s1, Figure S1: Total energy; Figure S2: Film thickness; Figures S3–S7: Density profiles; Figure S8: Convergence evidence; Figure S9: Interlayer distances; Figure S10: RDF between rGO-PSS; Figure S11: Density profile; Figure S12: Selective metric; Figure S13: RDF between water-film vs. methanol-film; Figure S14: RDF between hydronium-film; Figures S15 and S16: PSS model; Figure S17: rGO model; Figure S18: simulation box; Tables S1–S9: Force field parameters.

## Abbreviations

The following abbreviations are used in this manuscript:

rGO	Reduced Graphene Oxide
PSS	poly(styrenesulfonate)
DMFC	Direct Methanol Fuel Cell
MD	Molecular Dynamics
