# Supplementary material for: Computational Insights into Selective Water–Methanol Transport in rGO/PSS Composite Films
Source: Molecules. 2026 May 14;31(10):1657. doi: 10.3390/molecules31101657 (PMC13209839; doi:10.3390/molecules31101657)
Supplement: Supplementary file 1 [file molecules-31-01657-s001.zip › molecules-4202949-supplementary.pdf]

Supporting Information

Computational Insights into Selective Water–Methanol Transport in PSS/rGO Composite Films

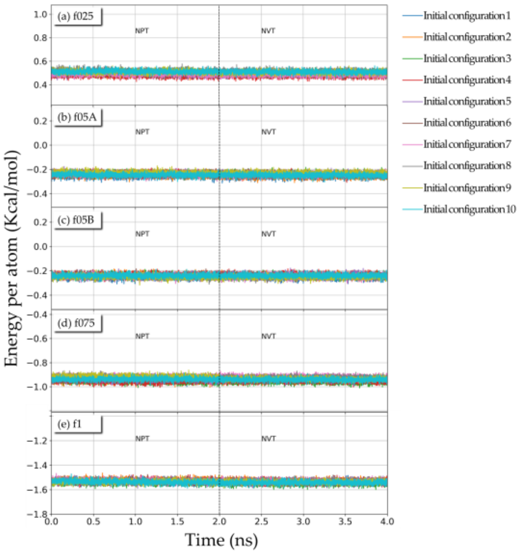

Figure S1. Total energy per atom over the course of the simulation for each rGO/PSS composition during the thermalization stage in the NPT ensemble and the production stage in the NVT ensemble.

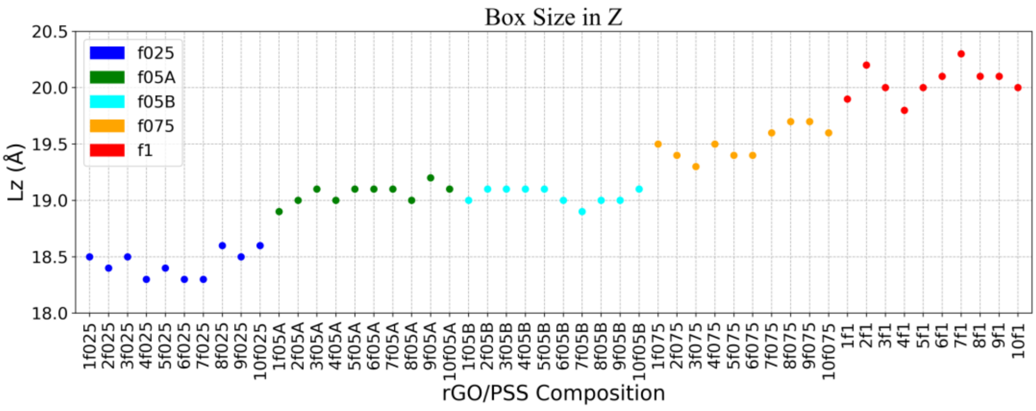

Figure S2. Box size in the Z-direction for each rGO/PSS composition.

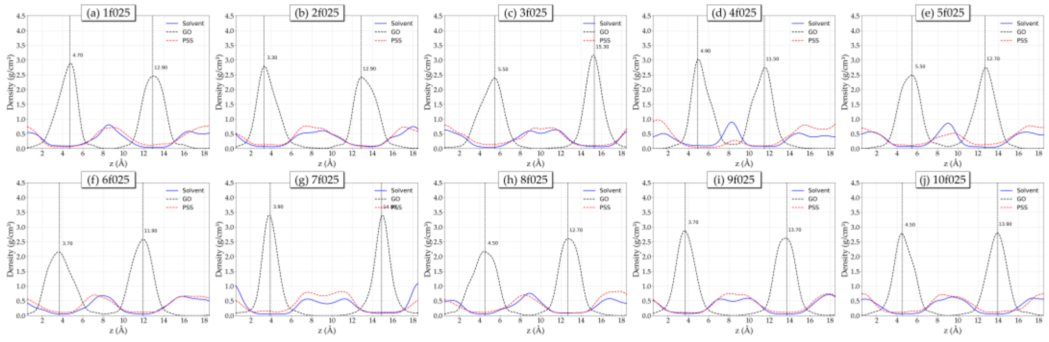

Figure S3. Mass density for each initial configuration for the rGO/PSS f025 composition.

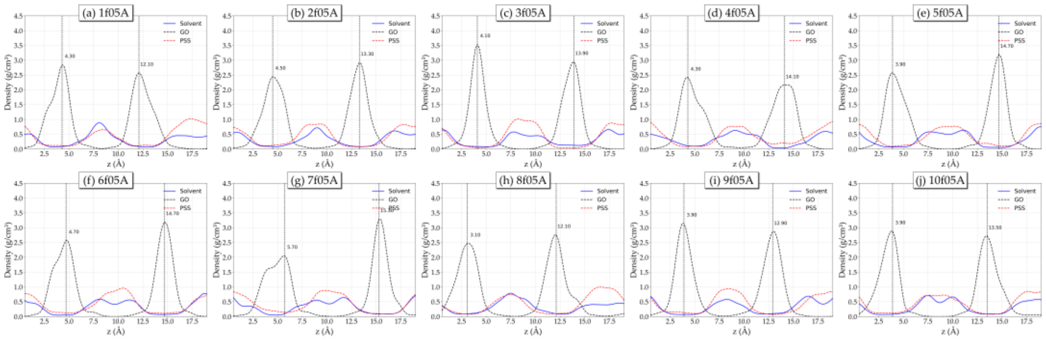

Figure S4. Mass density for each initial configuration for the rGO/PSS f05A composition.

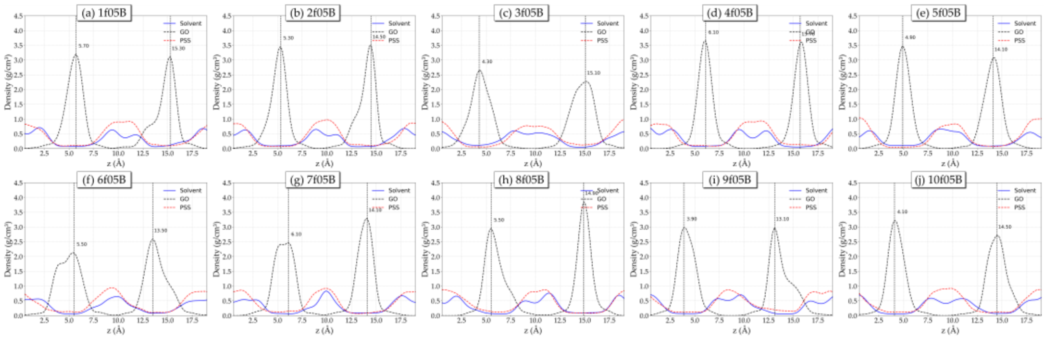

Figure S5. Mass density for each initial configuration for the rGO/PSS f05B composition.

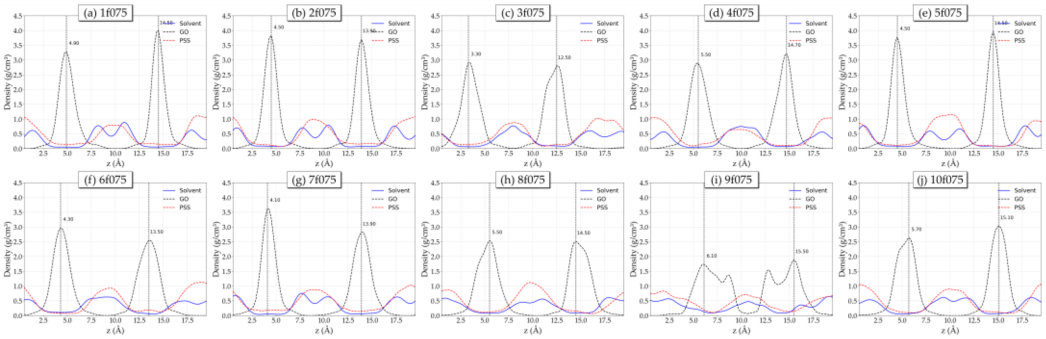

Figure S6. Mass density for each initial configuration for the rGO/PSS f075 composition.

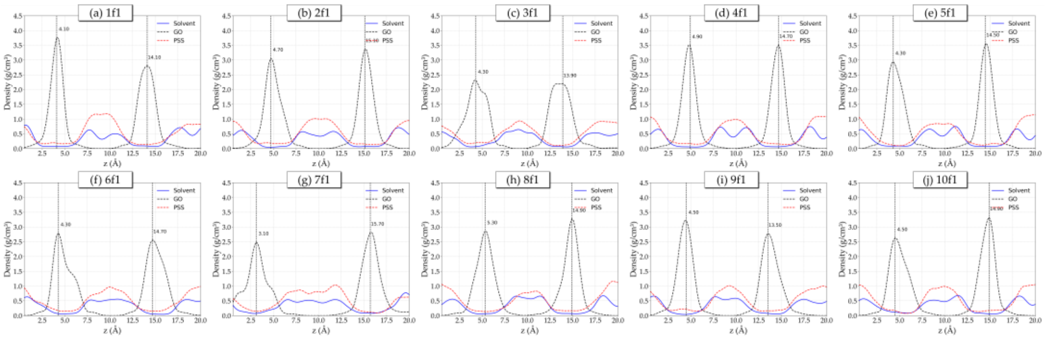

Figure S7. Mass density for each initial configuration for the rGO/PSS f1 composition.

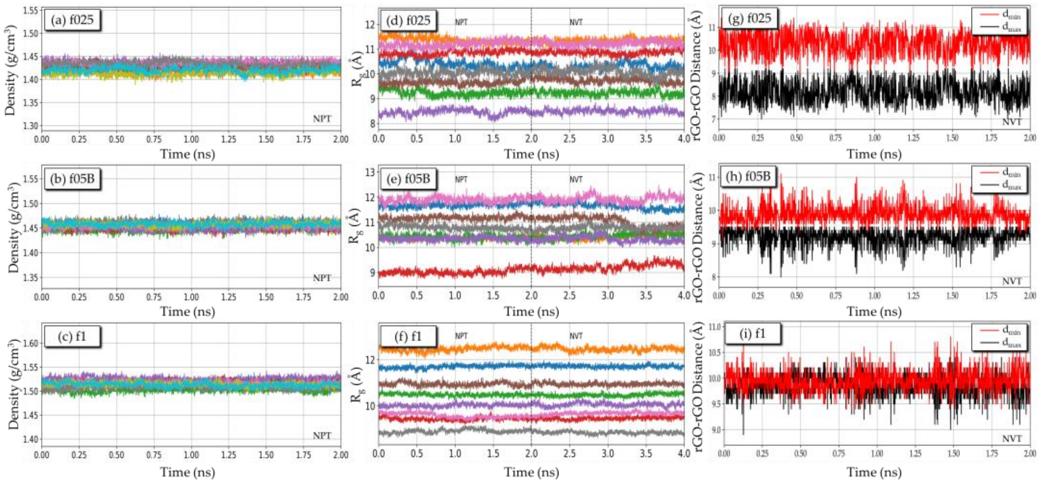

Figure S8. (a–c) Density, (d–f) radius of gyration, and (g–i) rGO–rGO distance as a function of time for the systems f025, f05B, and f1. These profiles confirm that the system does not undergo significant structural changes over the time interval encompassing both the NPT equilibration and subsequent NVT production phases.

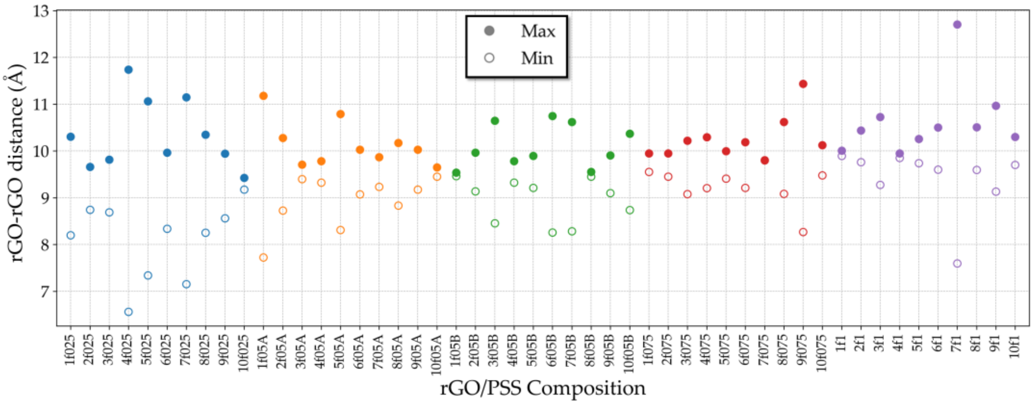

Figure S9. Maximum and minimum average rGO distance for each composition.

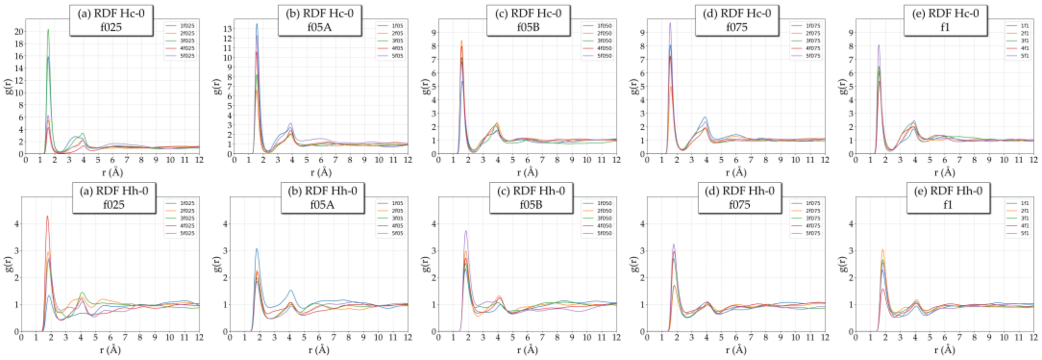

Figure S10. Radial distribution function between the carboxyl and hydroxyl groups of the rGO sheet with the oxygen atoms of PSS.

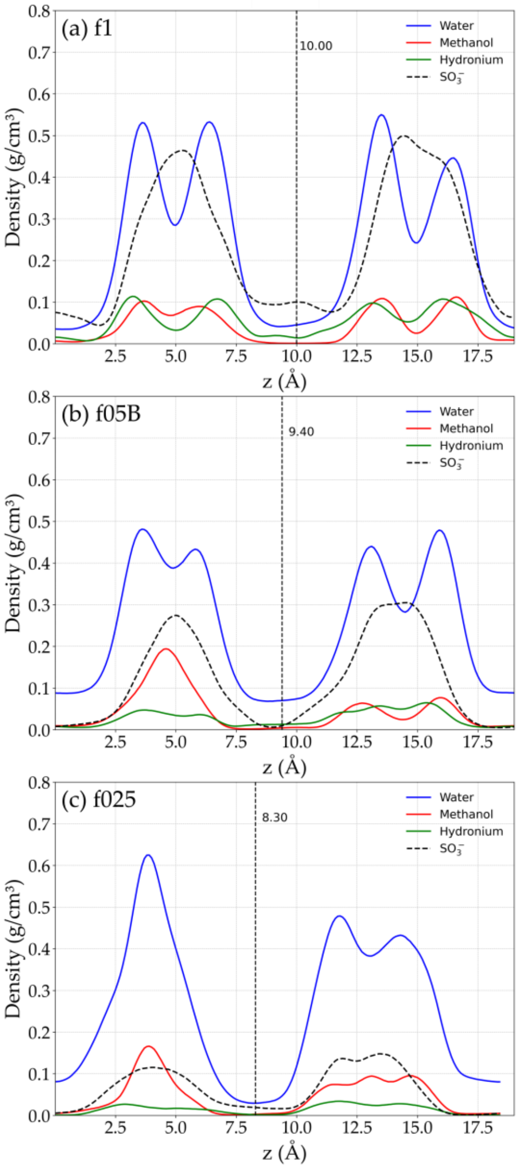

Figure S11. The mass profiles of water (blue), methanol (red), hydronium (green), and SO_3^- (black) for films with sulfonate fraction (a) f1 and (b) f05B and (c) f025, representing typical distribution of the components along the z-direction.

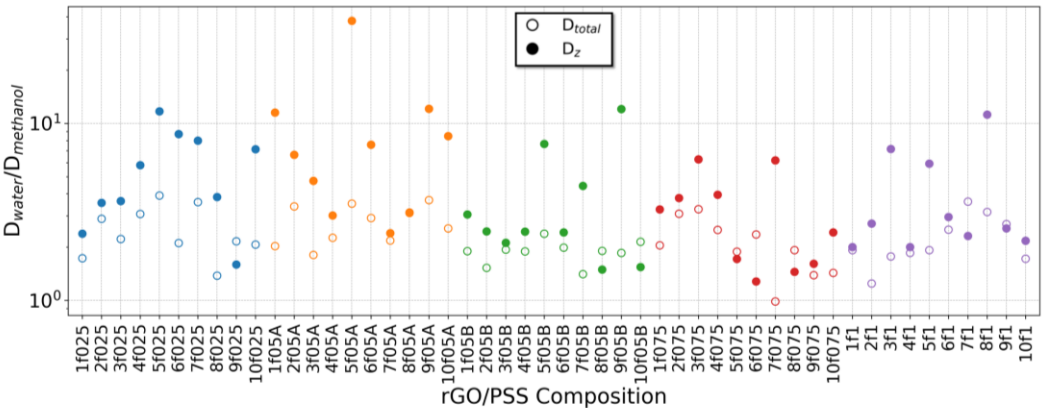

Figure S12. D(water)/D(methanol) ratio.

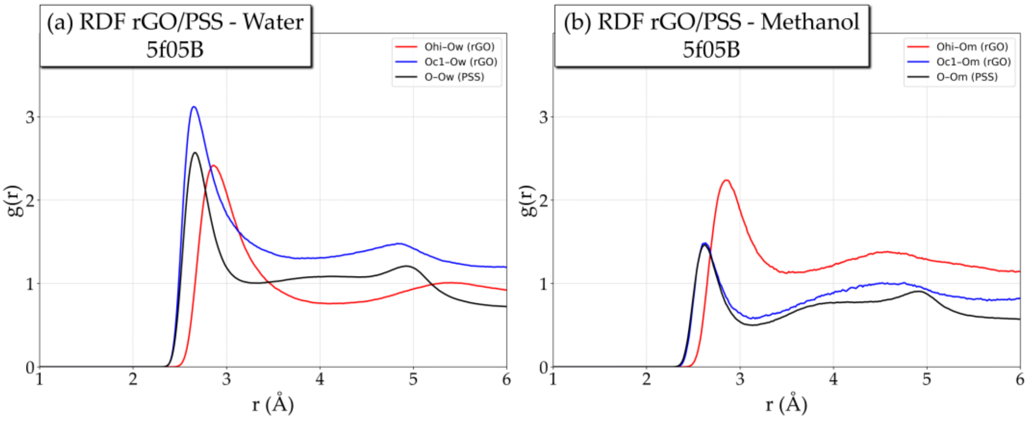

Figure S13. Radial distribution function between oxygen atoms of the carboxyl and hydroxyl groups of the rGO sheet and (a) water oxygen atoms and (b) methanol oxygen atoms.

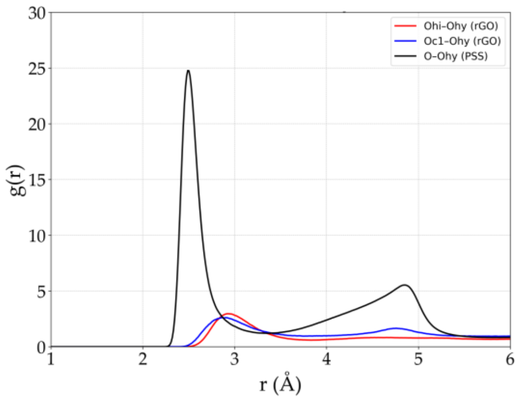

Figure S14. Radial distribution function between oxygen atoms of the carboxyl and hydroxyl groups of the rGO sheet and hydronium oxygen atoms.

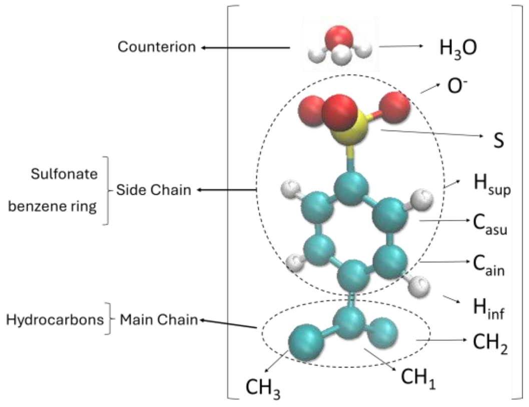

Figure S15. Diagram of the PSS monomer with hydronium counterion.

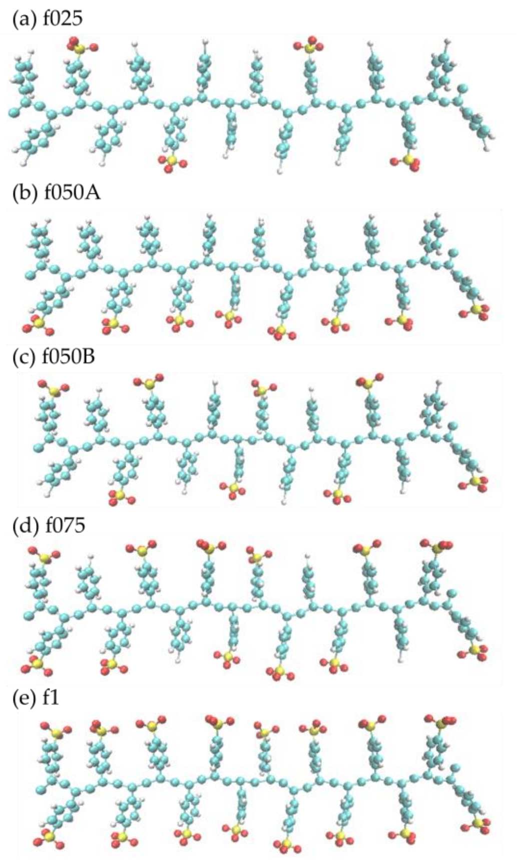

Figure S16. PSS configurations with different degrees of sulfonation.

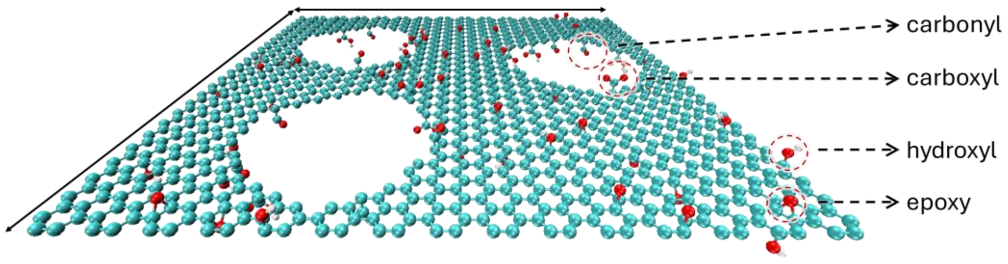

Figure S17. rGO sheet with oxygen-containing groups.

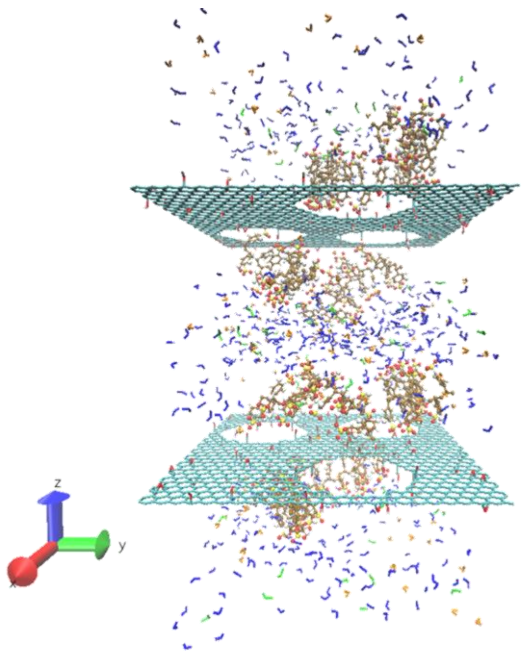

Figure S18. Initial configuration of GPSS (GPSS1F1). PSS carbon atoms are shown in ochre, water molecules in blue, methanol in green, hydronium in orange, and rGO carbon atoms in cyan.

Table S1. Lennard-Jones parameters and partial charges for PSS ($U_{LJ} = 4\epsilon[(\sigma/r)^{12} - (\sigma/r)^{6}]$).

LJ	ϵ (kcal/mol)	σ (Å)	Charge (e)
C_{ach}	0.0860	3.3997	-0.0556
C_{ao}	0.0860	3.3997	-0.1438
C_{ain}	0.0860	3.3997	-0.1133
C_{asu}	0.0860	3.3997	-0.0905
CH_1	0.0994	3.4887	0.0000
CH_2	0.1094	3.6669	0.0000
CH_3	0.1494	3.6669	0.0000
H	0.0150	2.5996	0.2670
H_{inf}	0.0150	2.5996	0.0631
H_{sup}	0.0150	2.5996	0.1069
S	0.3440	3.5903	1.1063
O	0.0957	3.0331	-0.6131
O_{hy}	0.1553	3.1600	-0.2480

Table S2. PSS bond parameters($U_b = K_b(r - r_o)^2$).

Bond	k_b (kcal/mol*Å^2)	r_0 (Å)
C_{ach}–C_{ain}	469	1.400
C_{ach}–CH_1	317	1.510
C_{ao}–C_{asu}	469	1.400
C_{ao}–H	367	1.080
C_{ao}–S	350	1.740

C_{ain}–C_{asu}	469	1.400
C_{ain} – C_{ain}	367	1.080
C_{ain} – H_{sup}	367	1.080
CH_1–CH_2	310	1.526
CH_1–CH_3	310	1.526
O–S	350	1.480
O_{hy}-H_{hy}	553.2	0.970

Table S3. PSS angle parameters ($U_\theta = K_\theta(\theta - \theta_o)^2$).

Angle	K_θ (kcal/mol · rad^2)	θ_0 (degrees)
C_{ach}–C_{ain}–C_{asu}	63.000	120.000
C_{ach}–C_{ain}–H_{inf}	35.000	120.000
C_{ach}–CH_1–CH_2	60.000	109.470
C_{ach}–CH_1–CH_3	60.000	109.470
C_{ao}–C_{asu}–C_{ain}	63.000	120.000
C_{ao}–C_{asu}–H_{sup}	35.000	120.000
C_{ao}–S–O	56.160	103.800
C_{ain}–C_{ach}–C_{ain}	63.000	120.000
C_{ain}–C_{ach}–CH_1	70.000	120.000
C_{ain}–C_{asu}–H_{sup}	35.000	120.000
C_{asu}–C_{ao}–C_{asu}	63.000	120.000
C_{asu}–C_{ao}–H	35.000	120.000

Table S4. PSS dihedral parameters ($U_\varphi = K_\varphi[1 + cos(n\varphi - d)]$).

Dihedral	$k\varphi$ (cal/mol)	n	d (degress)
C_{ach}–C_{ain}–C_{asu}–C_{ao}	3.1	2	180
C_{ach}–C_{ain}–C_{asu}–H_{sup}	3.1	2	180
C_{ach}–CH_1–CH_2–CH_1	1.4	3	0
C_{ain}–C_{ach}–C_{ain}–C_{asu}	3.1	2	180
C_{ain}–C_{ach}–C_{ain}–H_{inf}	3.1	2	180
C_{ain}–C_{ach}–CH_1–CH_2	0	0	0
C_{ain}–C_{ach}–CH_1–CH_3	0	0	0
C_{asu}–C_{ao}–C_{asu}–C_{ain}	3.1	2	180
C_{asu}–C_{ao}–C_{asu}–H_{sup}	3.1	2	180
C_{asu}–C_{ao}–S–O	1.3	2	180
CH_1–C_{ach}–C_{ain}–C_{asu}	3.1	2	180
CH_1–C_{ach}–C_{ain}–H_{inf}	1.4	3	0
CH_2–CH_1–CH_2–CH_1	1.4	3	0
CH_3–CH_1–CH_2–CH_1	1.4	3	0
H–C_{ao}–C_{asu}–C_{ain}	3.1	2	180
H–C_{ao}–C_{asu}–H_{sup}	3.1	2	180
H_{inf} –C_{ain}–C_{asu}–C_{ao}	3.1	2	180
H_{inf} –C_{ain}–C_{asu}–H_{sup}	3.1	2	180

$S–C_{ao}–C_{asu}–C_{ain}$	3.1	2	180
$S–C_{ao}–C_{asu}–H_{sup}$	0	0	0

Table S5. PSS improper dihedral parameters $(U_x = K_x(x - x_o)^2)$.

Improper	$K_x(Kcal.rad^{-2}.mol^{-1})$	$x\ (degress)$
$CH_1 - C_{ach} - C_{ain} - C_{ain}$	2	180

Table S6. Lennard-Jones parameters and partial charges for rGO $(U_{LJ} = 4\epsilon[(\sigma/r)^{12} - (\sigma/r)^6])$.

LJ	ϵ (kcal/mol)	σ (Å)	Charge (e)
C	0.0860	3.3997	0.0000
C_{ca1}	0.1066	3.7500	0.5500
C_{ca2}	0.0693	3.3997	0.0800
C_{ep}	0.0694	3.3997	0.2000
C_{hi}	0.0694	3.3997	0.1966
C_k	0.1050	3.7500	0.4610
H_{ca}	0.0000	0.0000	0.4500
H_{hi}	0.0000	0.0000	0.3294
O_{1ca}	0.1726	3.0000	-0.5800
O_{2ca}	0.2132	2.9600	-0.5000
O_{ep}	0.1422	3.0330	-0.4000
O_{hi}	0.1399	3.1660	-0.5260
O_k	0.2100	2.9600	-0.4610

Table S7. rGO bond parameters $(U_b = K_b(r - r_o)^2)$.

Bond	k_b (kcal/mol*Å^2)	r_0 (Å)
$C–C$	469.000	1.400
$C–C_{2c}$	469.000	1.400
$C–C_e$	365.000	1.502
$C–C_h$	365.000	1.502
$C–C_k$	254.000	1.460
$C_{1c}–C_{2c}$	300.000	1.480
$C_{1c}–O_{1c}$	230.000	1.400
$C_{1c}–O_{2c}$	750.000	1.220
$C_e–C_e$	222.500	1.523
$C_e–O_e$	220.000	1.450
$C_h–O_h$	428.000	1.420
$C_k–O_k$	700.000	1.230
$C_m–O_m$	1000.000	1.430
$H_c–O_{1c}$	545.000	0.960
$H_h–O_h$	545.000	0.960
$H_{hy}–O_{hy}$	553.285	0.970

H_m–O_m	1000.000	0.945
H_w–O_w	1000.000	1.000
O–S	350.000	1.480

Table S8. rGO angle parameters $(U_\theta = K_\theta(\theta - \theta_o)^2)$.

Angle	K_θ (kcal/mol · rad^2)	θ_0 (degrees)
C–C–C	63.000	120.000
C–C–C_{2c}	63.000	120.000
C–C–C_e	70.000	120.000
C–C–C_h	70.000	120.000
C–C–C_k	63.000	120.000
C–C_{2c}–C	63.000	120.000
C–C_{2c}–C_{1c}	85.000	120.000
C–C_e–C	40.000	109.500
C–C_e–C_e	63.000	114.000
C–C_e–O_e	50.000	109.500
C–C_h–C	40.000	109.500
C–C_h–O_h	50.000	109.500
C–C_k–O_k	70.000	121.300
C_{1c}–O_{1c}–H_c	35.000	113.000
C_{2c}–C–C_h	63.000	120.000
C_{2c}–C_{1c}–O_{1c}	70.000	120.000
C_{2c}–C_{1c}–O_{2c}	80.000	120.400
C_e–C–C_e	63.000	120.000
C_e–C–C_h	63.000	120.000
C_e–C_e–O_e	50.000	109.500
C_e–O_e–C_e	60.000	109.500
C_h–C–C_h	63.000	120.000
C_h–O_h–H_h	55.000	108.500
O_{1c}–C_{1c}–O_{2c}	80.000	121.000

Table S9. rGO dihedral parameters $(U_\varphi = K_\varphi[1 + cos(n\varphi - d)])$.

Dihedral	$k\varphi$ (cal/mol)	n	d (degress)
C–C–C–C	3.100	2	180
C–C–C–C_{2c}	0.500	1	180
C–C–C–C_e	3.100	2	180
C–C–C–C_h	3.100	2	180
C–C–C–C_k	3.100	2	180
C–C–C_{2c}–C	0.560	1	180
C–C–C_{2c}–C_{1c}	0.560	1	180
C–C–C_e–C	0.230	2	180

$C–C–C_e–C_e$	0.230	2	180
$C–C–C_h–C$	0.230	2	180
$C–C–C_k–O_k$	1.400	2	180
$C_{2c}–C–C–C_e$	3.100	2	180
$C_{2c}–C–C–C_h$	3.100	2	180
$C_{2c}–C–C_h–C$	3.100	2	180
$C_{2c}–C–C_h–O_h$	3.100	2	180
$C_{2c}–C_{1c}–O_{1c}–H_c$	0.975	1	180
$C_e–C–C–C_e$	3.100	2	180
$C_e–C–C–C_h$	3.100	2	180
$C_e–C–C–C_k$	3.100	2	180
$C_e–C–C_e–C$	3.100	2	180
$C_e–C–C_e–C_e$	3.100	2	180
$C_e–C–C_h–C$	3.100	2	180
$C_h–C–C–C_h$	3.100	2	180
$C_h–C–C–C_k$	3.100	2	180
$C_h–C–C_{2c}–C$	3.100	2	180
$C_h–C–C_{2c}–C_{1c}$	0.560	1	180
$C_h–C–C_e–C$	3.100	2	180
$C_h–C–C_e–C_e$	3.100	2	180
$C_h–C–C_h–C$	3.100	2	180
$O_{1c}–C_{1c}–C_{2c}–C$	1.400	2	180
$O_{2c}–C_{1c}–C_{2c}–C$	1.400	2	180
$O_{2c}–C_{1c}–O_{1c}–H_c$	2.050	2	180
